# TUBB3 overexpression has a negligible effect on the sensitivity to taxol in cultured cell lines

**DOI:** 10.18632/oncotarget.17740

**Published:** 2017-05-10

**Authors:** Mihoko A. Tame, Anna G. Manjón, Daria Belokhvostova, Jonne A. Raaijmakers, René H. Medema

**Affiliations:** ^1^ Division of Cell Biology and Cancer Genomics Center, The Netherlands Cancer Institute, 1066 CX Amsterdam, The Netherlands

**Keywords:** taxol, resistance, microtubule, βIII-tubulin, CRISPRa

## Abstract

Microtubules are cellular targets for a variety of anticancer therapies because of their critical function in mitosis. Taxol belongs to a class of microtubule targeting agents that suppresses microtubule dynamics and interferes with the functioning of the mitotic spindle, thereby effectively blocking cell cycle progression of rapidly proliferating tumor cells. Despite its antitumor activity, drug resistance remains a common obstacle in improving its overall clinical efficacy. Previous studies have shown that the expression of a specific β-tubulin isotype, βIII-tubulin/TUBB3, is dysregulated in drug-refractory tumors. However, whether enhanced TUBB3 expression is directly involved in promoting taxol resistance remains a subject of debate. Here, we have used several approaches to assess the functional relation of TUBB3 overexpression and taxol resistance. First, we generated a number of taxol-resistant cell lines, to find that TUBB3 expression was elevated in a resistant cell line (RPE-20) derived from untransformed retinal pigment epithelial (RPE) cells, but the abundance of TUBB3 remained unchanged in four other cell lines after taxol treatment. However, although RPE-20 cells displayed enhanced TUBB3 levels, we find that simultaneous up-regulation of the P-glycoprotein (P-gP) drug-efflux pump is responsible for the resistance to taxol. Indeed, we could show that TUBB3 levels were dynamically regulated upon taxol exposure and withdrawal, unrelated to the resistance phenotype. Next, we generated cell lines in which we could induce robust overexpression of TUBB3 from its endogenous locus employing the CRISPRa system. We demonstrate that solely enhancing TUBB3 expression results in a very minor decrease in the sensitivity to taxol. This was further substantiated by selective depletion of TUBB3 in a series of breast cancer cell lines expressing high levels of TUBB3. We find that TUBB3 depletion had a minimal effect on the sensitivity to taxol in one of these cell lines, but had no effect in all of the others. Based on these findings we propose that TUBB3 overexpression can only marginally affect the sensitivity to taxol in cultured cell lines.

## INTRODUCTION

Microtubules, polymers of α/β heterodimers, are dynamic cytoskeletal structures that are essential for many cellular functions, including cell movement, intracellular transport and cell division. Particularly during cell division, cells depend on the formation of a highly dynamic microtubule network, the mitotic spindle, which facilitates faithful segregation of chromosomes to the two new daughter cells [1]. Since uncontrolled cycles of cell divisions and chronic cell proliferation is a hallmark of many cancers [2], microtubules (MTs) have been exploited as therapeutic targets to curb proliferation of transformed cells using a variety of microtubule-targeting agents (MTAs), also known as anti-mitotics [3].

Paclitaxel (hereafter referred to as taxol) is an MTA that suppresses microtubule dynamics and thereby disrupts mitotic progression. This mode of action is thought to be responsible for the potent ability of taxol to prevent cell proliferation in tumors [4, 5]. Taxol is used for the treatment of a variety of solid tumors, such as ovarian, breast and lung cancers [6]. However, in spite of its initial antitumor activity, the overall clinical efficacy of this drug is often limited due to intrinsic or acquired drug resistance [3, 7]. Determining molecular mechanisms of taxol resistance is therefore of great clinical value for the design of treatment plans.

Taxol specifically targets the β-subunit of tubulin [6], of which eight isotypes exist in humans [8]. The β-tubulin isotypes are highly conserved in their core globular domain; however they display subtle differences in their unstructured C-terminal tails, a region of the protein that is positioned at the exterior surface of the polymerized MT lattice and provides sites for a variety of post-translational modifications as well as binding sites for microtubule-associated proteins [9, 10]. Expression of most of the β-tubulin isotypes is confined to specific cell types or tissues, and certain compositions of tubulin isotypes may assemble into discrete MT species with unique properties and functions [11, 12].

Interestingly, tumors that have become refractory to taxol treatment frequently express different sets of β-tubulin isotypes that are not expressed in their tissue of origin. In particular, the selective over-expression of class III β-tubulin (TUBB3) has been reported to be associated with taxol resistance in an overwhelming number of translational studies (reviewed in [13, 14]). Functional studies subsequently corroborated a direct role of TUBB3 in enhancing taxol resistance. TUBB3 knockdown in cancer cell lines that have aberrantly high expression of this gene product were shown to result in increased sensitivity to taxol [15–17], whereas ectopic over-expression of this gene in cell lines with low basal expression level of TUBB3 is accompanied by increased resistance to taxol [18, 19]. Furthermore, *in vitro* studies demonstrated that TUBB3 enhances the rate of tubulin depolymerization in the presence of taxol [18, 20, 21], indicating that TUBB3 overexpression might directly render microtubules less sensitive to the MT-stabilizing activity of taxol. Based on these studies, the overexpression of TUBB3 has been initially considered as a promising predictive marker for taxol resistance in tumors.

However, several other studies have since then implicated a broader function for TUBB3 in drug resistance or as a general cell survival factor. For instance, increased expression of TUBB3 confers cells with resistance to other chemotherapeutic drugs, including vinca alkaloids and DNA damaging agents [15, 22]. Furthermore, TUBB3 overexpression has been observed upon exposure of cells to challenging growth conditions, such as nutrient deprivation [23] and hypoxia [24]. Moreover, increased expression of TUBB3 has been associated with aggressive tumor phenotypes in patients that have never been treated with taxol-containing regimens (reviewed in [25]).

In this study, we addressed the regulation and functional significance of TUBB3 in taxol resistance with multiple different experimental set-ups and a variety of cell lines. We have identified in multiple incidences a correlation between taxol sensitivity and increased TUBB3 expression. However, although induced overexpression of TUBB3 is sufficient for a minor taxol-resistance phenotype, TUBB3 depletion experiments show that it has no major role in driving drug resistance, therefore, other b-isotypes may contribute to this process. Our work highlights the multifactorial nature of taxol resistance in cultured cell lines, and shows that TUBB3 overexpression in untransformed cells has a very minor effect on the taxol sensitivity.

## RESULTS

### Taxol-resistance of RPE-20 is mediated through P-gP

**Figure 1:**
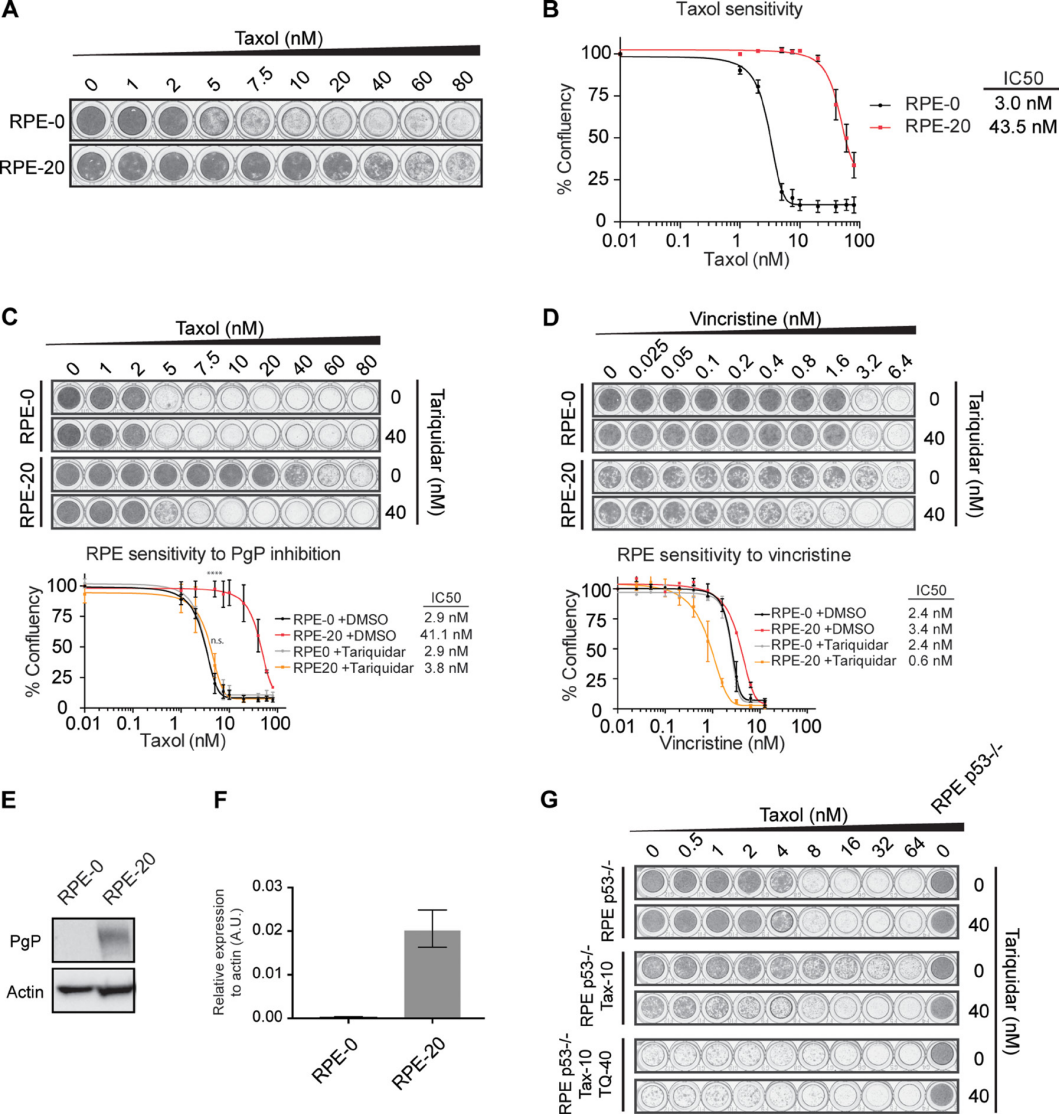
Taxol-resistance in RPE-20 is predominantly mediated through the up-regulation of the P-gP drug pump (**A**) Crystal violet staining of viability assay with taxol-naïve RPE-0 and resistant RPE-20 cell lines. (**B**) Relative survival plots of the RPE-0 and RPE-20 cell lines. Shown are the average +/− s.d. of three independent experiments and the calculated IC50. (**C**) Relative survival plots of the same cell lines as in A) and B) in an increasing dose of taxol and 0 and 40 nM of Tariquidar. ANOVA Turkey's multiple comparisons test. Graph shows mean +/− SEM. (****P < 0.0001). (**D**) Relative survival plots of RPE-0 and RPE-20 cells in an increasing dose of vincristine and 0 and 40 nM of Tariquidar. For all conditions, viability assays were carried out by growing ∼1000 cells for 7 days. (**E**) Western blot showing increased levels of P-gP in the taxol-resistant RPE-20 cell line compared to RPE-0. (**F**) P-gP mRNA levels were determined by qRT-PCR. Values were normalized to actin expression levels. Error bars are obtained from experimental triplicates. (**G**) Relative survival plots with a drug-naïve RPE p53−/−, a taxol-resistant RPE p53−/− (Tax-10), and a taxol-resistant RPE p53−/− cell line that was generated by a combined treatment with 40 nM of the P-gP inhibitor (Tax-10, TQ-40).

We generated taxol-resistant cell lines derived from hTERT-immortalized, untransformed RPE-1 (RPE) cells through prolonged exposure and clonogenic outgrowth in the presence of an increasing dose of taxol. After polyclonal selection of taxol-resistant cells for at least 4 weeks, we obtained a cell line that could proliferate under constant exposure to 20 nM of taxol (RPE-20) (Figure 1A). In terms of IC50, the RPE-20 cell line displayed a 14-fold increased resistance to taxol compared to the parental counterpart (RPE-0) (Figure 1B; IC50 = 3.0 for RPE-0, IC50 = 43.5 for RPE-20). A predominant mechanism of taxol resistance reported in studies utilizing cultured cell lines is the up-regulation of the drug efflux pump P-glycoprotein (P-gP)/ABCB1 (reviewed in [26]). Thus, we decided to first test if taxol resistance in the RPE-20 cells is mediated through P-gP. Relative survival plots revealed that RPE-20 cells became highly sensitive to taxol when treated in combination with tariquidar, a specific inhibitor of P-gP [27]. While the RPE-20 cells have an IC50 for taxol of 41.1 nM in the absence of the inhibitor, their resistance dropped to an IC50 of 3.8 nM after tariquidar addition, similar to the IC50 for the parental RPE cells (Figure 1C). This result suggests that an increased efflux of the drug mediated by P-gP predominantly facilitates taxol resistance in the RPE-20. Furthermore, these cells display cross-resistance to vincristine (Figure 1D), an MTA that is also a well-described substrate of P-gP [26]. In line with this idea, we confirmed that RPE-20 cells express increased amount of P-gP both in protein (Figure 1E) and mRNA level (Figure 1F). In an attempt to establish a P-gP-independent taxol-resistant RPE cell line, we cultured RPE cells in the presence of 5 nM taxol and 40 nM of tariquidar. However, this approach did not yield any surviving clones (data not shown). Furthermore, we repeated the same approach with a p53-deficient RPE cell line. Although RPE p53−/− cells grew out resistant colonies and were viable after increasing the dose of taxol to 10 nM, their proliferation was severely reduced in the presence of tariquidar (Figure 1G). Thus, this suggests that P-gP is an important driver of taxol resistance in RPE cells and their proliferative capacity is severely compromised when forced to adapt to taxol through alternative mechanisms. Nonetheless, we observed that the RPE-20 cells remain slightly more resistant to taxol even in the presence of tariquidar (IC50 = 3.8 nM) compared to the RPE-0 cells (IC50 = 2.9 nM) (Figure 1C). Moreover, RPE-20 cells were hypersensitive to the MT-destabilizing drug vincristine, when treated in combination with tariquidar (Figure 1D). These results suggest that while the induction of P-gP activity provides the major mechanism of taxol-resistance in RPE cells, they may have also adapted their MT dynamics to the stabilizing effect of taxol, albeit that the contribution of the altered MT dynamics to the overall sensitivity to taxol appears to be very minor.

### TUBB3 protein levels are dynamically regulated upon taxol exposure and withdrawal and does not correlate with the timing of resistance acquisition

**Figure 2:**
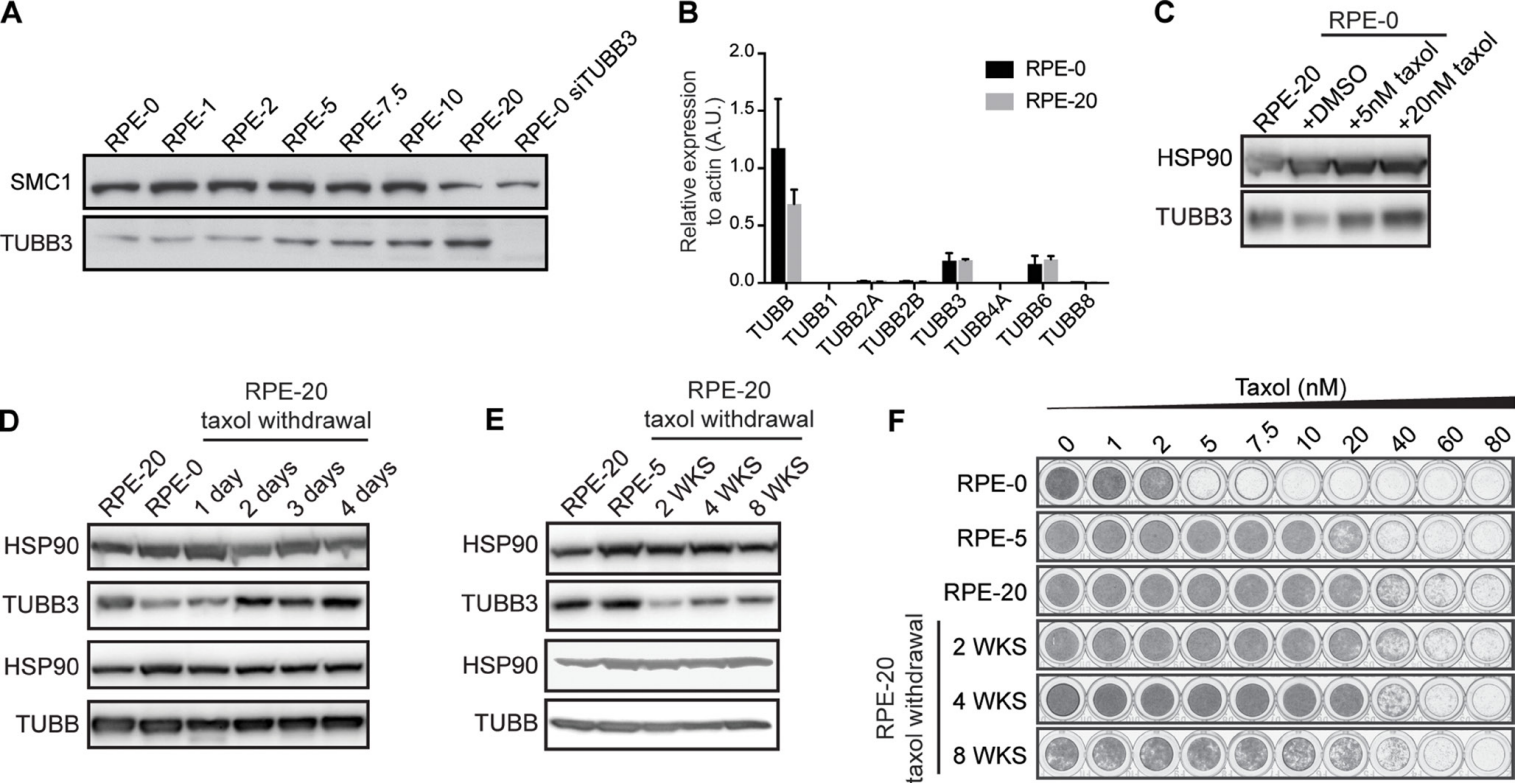
TUBB3 levels are dynamically regulated in RPE cells upon taxol exposure and withdrawal (**A**) Western blot showing TUBB3 levels in cell lysates prepared from taxol-resistant RPE cells. Taxol-naïve RPE cells (RPE-0) exhibit low basal levels of TUBB3. Note that RPE-1 and RPE-2 indicate cell lines that have been continuously cultured in the presence of 1 and 2 nM of taxol, respectively, while cell proliferation and viability of RPE cells was not visibly affected at these drug concentrations (see Figure 1A)). The RPE-5, −7.5, −10, and −20 are cell lines derived from polyclonal selection of resistant cells that have survived taxol treatment over a drug selection period of 4-6 weeks. (**B**) b-tubulin isotypes mRNA levels were determined by qRT-PCR. Values were normalized to actin expression levels. Error bars are obtained from two independent experiments. (**C**) Western blot showing the rapid induction of TUBB3 levels after a short-term, 30-hour treatment of RPE-0 cells with 5 and 20 nM of taxol. (**D**) Fluctuating TUBB3 levels immediately after taxol withdrawal from the resistant RPE-20 cells and a further reduction of TUBB3 levels observed after up to 8 weeks of taxol-withdrawal in (**E**, **F**) Viability assays were performed with the RPE-20 cells after different periods of taxol withdrawal, corresponding to the time-points examined in E).

Next, we set out to examine whether TUBB3 levels are altered in the RPE-20 cells compared to the taxol-naïve RPE cells to account for the minor decrease in taxol-sensitivity that we observed in the presence of tariquidar (Figure 1C). Surprisingly, we observed an increase in TUBB3 protein levels in taxol-resistant RPE cells compared to control DMSO-treated cells (Figure 2A), similar to what was observed previously in the A549-T24 non-small-cell lung cancer [16] and DU-145 prostate carcinoma cells [28]. We confirmed the specificity of the TUBB3 antibody by western blotting of cell lysates collected after siRNA-mediated knockdown of this protein (Figure 2A). Continuous exposure of RPE cells to a dose of taxol at which cell proliferation is not affected (up to 2 nM, Figure 1A and B) did not affect the expression level of TUBB3 (Figure 2A). Next, we conducted siRNA-mediated knockdown of βIII-tubulin to assess its role in the resistance of the RPE-20 cell line. Two of our siRNAs targeting TUBB3 displayed strong anti-proliferative effects (Supplementary Figure 1A), but a third (siTUBB3 #9) achieved an equally efficient knock-down of TUBB3, without affecting cell proliferation, indicating that siRNAs #6 and #8 induce off-target effects, whereas #7 induces a relatively mild depletion. Using siTUBB3 #9, we achieved almost complete TUBB3 knockdown (Supplementary Figure 1A), but the sensitivity of RPE-20 to taxol was unchanged as assessed by viability assays (Supplementary Figure 1B). Hence, we conclude that βIII-tubulin has no role in the taxol-resistance of the RPE-20 cells.

Nonetheless, taxol affected TUBB3 levels in a dose-dependent manner (Figure 2A). In order determine whether other β-tubulin isotypes where up- or down-regulated in RPE-20, we performed qRT-PCRs to examine the mRNA levels of other isotypes (Figure 2B). Interestingly, we observed a similar expression profile in RPE-20 compared to RPE-0, indicating that the elevated levels of TUBB3 protein are not due to transcriptional up-regulation. We next performed Mass-spectrometry-based quantitative proteomics in RPE-20 to investigate the protein levels of various β-tubulin isotypes (Supplementary Figure 2A). We find that expression of TUBB3 is most prominently increased, but also observe more modest increases in expression of TUBB4A/βIV-tubulin and TUBB6/βV-tubulin, whereas expression of TUBB/βI-tubulin is somewhat decreased. This indicates that several β-tubulin isotypes are stabilized in the taxol-resistant RPE-20. However, given the primary role for P-gP in the observed resistance (Figure 1), we can conclude that these changes have very limited effects on the overall response to taxol.

To test whether TUBB3 overexpression is induced in other cell lines selected for taxol-resistance, we generated taxol-resistant cell lines derived from a colorectal carcinoma (HCT116), an osteosarcoma (U2OS), and two triple-negative breast cancer cell lines (Cal-51 and HCC1806). After polyclonal selection of taxol-resistant cells, we obtained cell lines that could tolerate at least twice the dose of taxol when compared to their parental counterparts (Supplementary Figure 3A). Next, we examined the levels of TUBB3 expression in these resistant cell lines and found no altered TUBB3 levels in the four taxol resistant cancer cell lines relative to their respective parental cell lines (Supplementary Figure 3B). Thus, although some cell lines exhibit elevated levels of TUBB3 upon selection with taxol, as was observed with the RPE-20 cell line in this study and a number of other cancer cell lines in other studies [16, 28, 29], this is by no means a phenomenon that occurs ubiquitously.

We further examined TUBB3 regulation after exposure of taxol-naïve RPE cells to this drug for a short period of time. Surprisingly, we observed an increase of TUBB3 levels relative to control cells after 30 hours of taxol at concentrations of 5 and 20 nM, respectively (Figure 2C). Inversely, we observed a rapid reduction in TUBB3 abundance, to a level comparable to taxol-naïve cells, after one day of removing taxol from the culture medium of RPE-20 cells (Figure 2D). A low level of TUBB3 was maintained after prolonged taxol withdrawal of up to eight weeks (Figure 2E). Relative survival plots conducted in parallel showed that while TUBB3 levels are reduced after taxol withdrawal, cells remained as resistant to taxol as the RPE-20 cells (Figure 2F). These rapid and reversible changes in TUBB3 levels occurring after taxol treatment of RPE cells (Figure 2C), and taking into account that the mRNA levels in RPE-20 remain similar to RPE-0 (Figure 2B), indicate that TUBB3 protein stabilization is dynamically regulated. This same trend could also happen with particular isotypes like TUBB, TUBB4A or TUBB6, but more work is required to resolve this. Altogether, this may indicate part of a more general cellular response to stress, analogous to TUBB3 up-regulation observed after exposure of cells to toxic microenvironments, such as hypoxia or nutrient deprivation [23, 24].

### Overexpression of TUBB3 in RPE cells plays a minor role in taxol resistance

**Figure 3:**
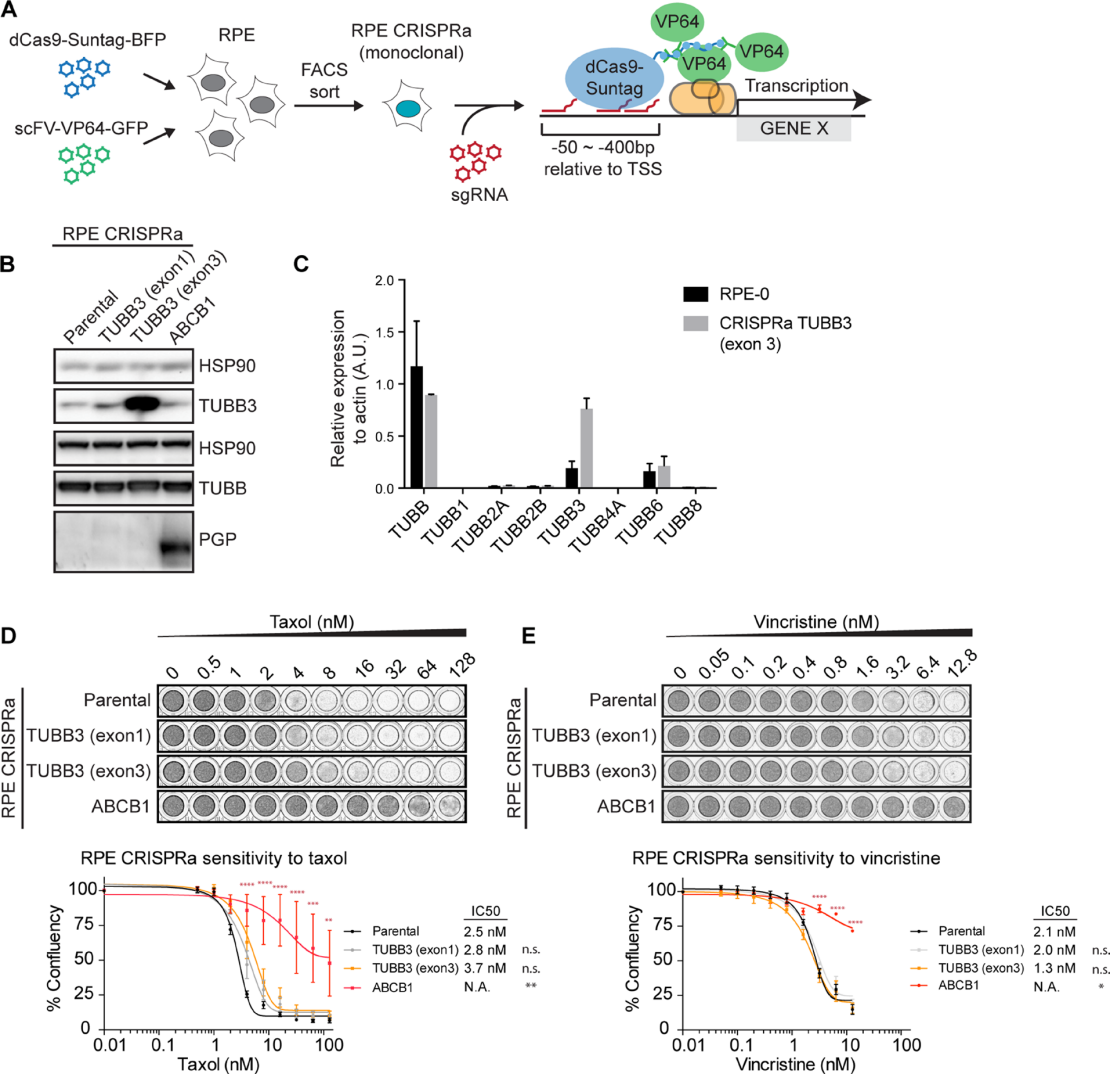
Overexpression of TUBB3 in RPE cells plays a minor role in taxol resistance (**A**) Schematic depicting the procedure for generating CRISPRa cell lines. (**B**) Western blots showing the expression levels of TUBB3 and P-gP after transduction of the CRISPRa cell lines with pools of sgRNAs targeted at putative enhancer regions of the respective genes. (**C**) b-tubulin isotypes mRNA levels were determined by qRT-PCR. Values were normalized to actin expression levels. Error bars are obtained from two independent experiments. (**D**) Relative survival plots of the TUBB3 and P-gP-overexpressing CRISPRa cell lines in increasing doses of taxol and (**E**) vincristine. ANOVA Turkey's multiple comparisons test. Graph shows mean +/− SEM. (****P < 0.0001, ***P < 0.001, **P < 0.01).

To further corroborate the notion that TUBB3 expression levels have a negligible effect on the sensitivity to spindle poisons like taxol and vincristine, we introduced the SunTag-Cas9 (CRISPRa) system in RPE cells, which allows specific and robust transcriptional activation of genes of interest through sgRNA-Cas9-mediated targeting of synthetic transcriptional activators to upstream regions of transcriptional start sites (TSS) (Figure 3A, [30, 31]). Examination of the TUBB3 gene locus in the USCS genome browser (https://genome.ucsc.edu/) revealed the presence of two prominent histone3 lysine27 acetylation (H3K27Ac)-rich regions, a type of histone modification known as a marker of active gene regulation [32]. The first H3K27Ac-rich region is located upstream of exon 1 and a second region is flanked by exon 2 and 3 of the TUBB3 gene locus. This indicates the presence of an intragenic enhancer for the transcriptional regulation of TUBB3, aside of a conventional enhancer at the 5′-UTR. Thus, we decided to design two separate sets of sgRNA pools, each targeting one of the H3K27Ac-rich regions (Supplementary Table 1). In addition to using this system for the transcriptional activation of TUBB3, we sought to generate a CRISPRa cell line for the activation of P-gP/ABCB1 as a positive control (Supplementary Table 1). We packaged the three pools of sgRNAs (sgTUBB3 exon 1, sgTUBB3 exon 3, and sgABCB1) into separate lentiviral particles and transduced monoclonal RPE cells stably expressing CRISPRa (Figure 3A).

By western blot analysis, we confirmed the specific induction of P-gP in the CRISPRa cell lines co-expressing sgRNAs targeted against ABCB1 (Figure 3B). As expected, P-gP over-expression (RPE CRISPRa sgABCB1) promoted a significant level of drug resistance against taxol (Figure 3D) as well as vincristine (Figure 3E). For TUBB3, we observed that the two distinct sgTUBB3 pools induced differential levels of TUBB3. While the expression of sgTUBB3 exon 1 induced a minor increase in TUBB3 protein levels, we achieved highly efficient over-expression of TUBB3 with the sgTUBB3 targeting upstream of exon 3 (Figure 3B). This over-expression was also confirmed by qRT-PCR, where the CRISPRa showed a ∼4-fold increase in TUBB3 mRNA levels (Figure 3C). Although the overall β-tubulin levels were comparable between the parental and the TUBB3 over-expressing cells (Figure 3B), none of the other β-tubulin isotypes were down-regulated or up-regulated at the mRNA levels (Figure 3C). Furthermore, mass-spectrometry experiments performed with CRISPRa TUBB3 (exon 3) show that the TUBB3 protein is the only isotype that is upregulated in these cells (Supplementary Figure 2B). Interestingly, relative survival plots revealed that the sensitivity of the TUBB3 over-expressing cell lines to taxol is very comparable to the parental cell line. The IC50 of RPE CRISPRa cells expressing sgTUBB3 exon 1 was 2.8 nM, a 1.1-fold increase compared to parental cells (IC50 of 2.5 nM), while those expressing sgTUBB3 exon 3 showed an IC50 of 3.7 nM (1.5-fold) (Figure 3D). Over-expression of TUBB3 also minimally altered the sensitivity of these cells to vincristine (Figure 3E). We therefore conclude that induced overexpression of TUBB3 is unable to promote a clear taxol resistance phenotype. Given the fact that we find that protein levels of none of the other detectable β-tubulin isotypes change (Supplementary Figure 2B), it is also unlikely that different expression of other β-tubulin isotypes affects taxol- resistance and sensitivity in CRISPRa TUBB3 cell lines.

### Differential functional requirement for TUBB3 in breast cancer cell lines

**Figure 4:**
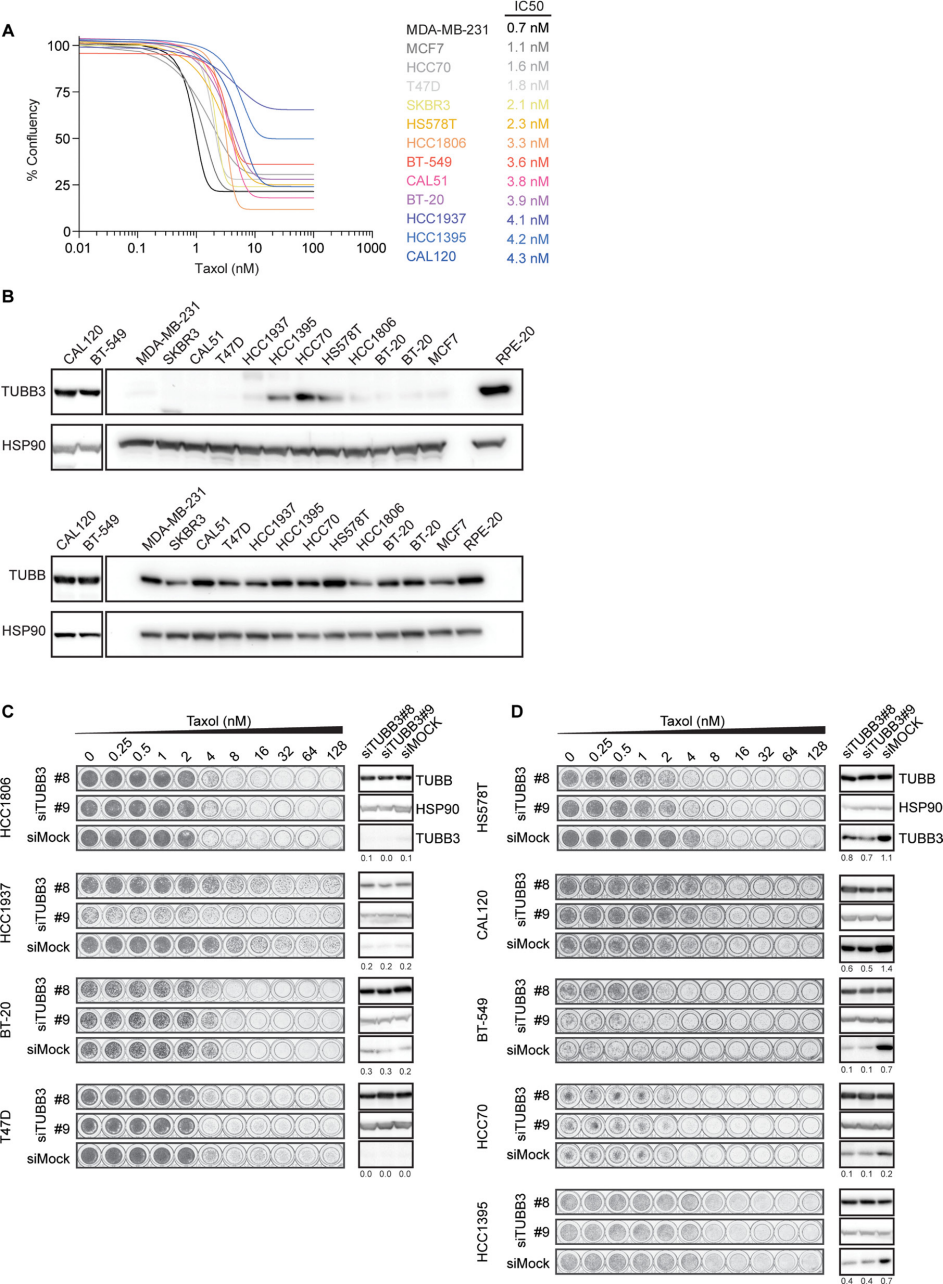
TUBB3 expression in breast cancer cell lines and taxol sensitivity (**A**) Sensitivity of breast cancer cell lines to taxol determined by relative survival plots with increasing concentrations of taxol for 7 days. Relative survival plots and IC50 were determined from at least three independent viability assays. For better visibility, the s.d. was excluded from this graph. (**B**) Western blots showing the TUBB3 (top) and overall β-tubulin levels (bottom) in the breast cancer cell lines. (**C**) Relative survival plots of four cell lines with low and (**D**) five cell lines with high levels of TUBB3 treated with different doses of taxol for 7 days after knockdown of TUBB3. Cells were transfected with siRNA targeted against TUBB3 for 48 hours prior to re-plating them into new plates containing an increasing concentration of taxol. Numbers below each TUBB3 blot indicate relative levels of TUBB3 normalized by loading controls.

Lastly, we set out to examine the functional relevance of TUBB3 overexpression in taxol resistance in a panel of breast cancer cell lines. We determined taxol sensitivity in 13 cell lines, most of which are triple negative breast cancer cells (Figure 4A, [33]). The panel of cell lines comprised a maximum ∼7-fold difference in taxol sensitivity between the most and least sensitive cell lines, with an IC50 of 0.7 nM up to 4.3 nM. Next, we examined TUBB3 levels and found relatively high expression of this protein in five of the cell lines (CAL120, BT549, HCC1395, HCC70, and HS578T), while TUBB3 was barely detectable in the remaining eight cell lines (Figure 4B). Interestingly, despite the limited sample size, comparison of TUBB3 levels with IC50 revealed a slight positive correlation (R^2^=0.06846) between these two factors (Supplementary Figure 4). To test whether there is a functional role of TUBB3 in conferring these cells with decreased sensitivity to taxol, we performed viability assays after TUBB3 depletion using two independent siRNAs. As expected, in control cell lines with low or undetectable levels of TUBB3 (HCC1806, HCC1937, BT20, and T47D), taxol sensitivity was unaffected after transfection of cells with siRNA targeted against TUBB3 as compared to Mock-depleted cells (Figure 4C). Similarly, in four out of the five cell lines that had high levels of TUBB3 (CAL120, BT549, HCC70, and HCC1395), depletion of this protein did not sensitize the cells to taxol (Figure 4D). In one cell line (HS578T), we observed a minimal but consistent increase in taxol sensitivity after depletion of TUBB3 with both siRNAs (Figure 4D), but again the enhancement of taxol sensitivity after TUBB3 depletion was minor. This suggests that TUBB3 overexpression has a very limited effect on sensitivity to taxol in certain cell types. Nonetheless, this functional role of TUBB3 is not generally applicable, as taxol treatment after TUBB3 depletion in the majority of cell lines tested has no significant effect on cell viability.

## DISCUSSION AND CONCLUSIONS

We have generated a number of taxol-resistant cell lines in culture and examined changes in their β-tubulin isotypes expression levels compared to their parental counterparts, specially focusing on TUBB3. While we observed no induction in TUBB3 expression in multiple cancer cell lines, we did detect a prominent increase in TUBB3 protein levels in taxol-resistant RPE cells. However, further analysis revealed that TUBB3 levels are dynamically regulated upon taxol-treatment in taxol-naïve RPE cells, and in response to taxol-withdrawal from the resistant RPE-20 cells. This regulation occurred unrelated to the timing of acquisition of the taxol-resistance phenotype. This observation makes it difficult to sustain the idea that deregulated TUBB3 overexpression is responsible for the acquisition of taxol-resistance of RPE-20 cells. Rather, the induction of TUBB3 levels appears to occur as a cellular response, perhaps analogous to what has been observed after exposure of cells to other types of cellular stress [23, 24]. Indeed, we find that the major mechanism of taxol-resistance in the RPE-20 cells can be attributed to the activity of P-gP drug efflux pump, a factor that frequently contributes to multi-drug resistance in cell culture [26].

In order to determine whether other β-tubulin isotypes could play a role in taxol resistance, we also assessed their expression levels in RPE-20. While we could not observe over-expression of any isotype at the transcriptional level, we found that TUBB3 protein was most prominently up-regulated, while expression of the TUBB4 and TUBB6 isotypes was slightly enhanced. On the other hand, TUBB levels appear to be slightly down-regulated, perhaps to compensate the increase of the other isotypes. Although TUBB3 is the most described isotype to play a role in taxol resistance, other β-tubulin classes, such as β-IV (TUBB4) and β-V (TUBB6) can also affect sensitivity to tubulin binding drugs [16, 34, 35]. While we focused our study in TUBB3 expression upon taxol exposure and withdrawal, it would seem that the changes in TUBB4 and TUBB6 expression observed in the RPE-20 cells also have little effect on taxol-sensitivity. Nonetheless, it would be interesting to test if mere expression of TUBB4 or TUBB6 at higher levels, like we did for TUBB3 using the CRISPRa-system, can affect the sensitivity to taxol.

As an alternative approach for the direct functional assessment of TUBB3 in chemotherapy resistance, we have further established an RPE cell line that efficiently over-expresses TUBB3 from its endogenous locus by utilizing the CRISPRa technology. Interestingly, CRISPRa-mediated recruitment of the transcriptional machinery to both the 5′-UTR as well as to an intragenic region flanking exons 2 and 3 induces enhanced expression of TUBB3. Under hypoxic conditions, the recruitment of hypoxia-induced transcription factors HIF-1α and HIF-2α to an E-box motif located at the 3′-UTR of the TUBB3 locus induces TUBB3 expression [24]. Although additional experiments are needed to determine the function and regulation of these two new putative enhancer regions, our data indicate that additional mechanisms of TUBB3 transcriptional regulation, aside of regulation by HIFs at the 3′-UTR, are likely to exist. It remains an interesting question for the future to identify transcription factors that regulate these sites.

Importantly, we show that CRISPRa-mediated TUBB3 over-expression leads to a very limited change in taxol sensitivity, which is in line with previous studies that failed to find a clear link between taxol-sensitivity and TUBB3 expression levels [18, 20, 21]. Furthermore, the overexpression of TUBB3 confers cells with minimally, but consistently increased sensitivity to the MT-destabilizing drug vincristine. Overexpression of this particular β-tubulin isotype may alter MT dynamics to counteract the activity of MT-stabilizing agents, while synergizing with MT-destabilizers. This is in line with the observation that microtubules assembled from TUBB3 exhibit increased dynamicity compared to microtubules composed of other β-tubulin isotypes [36–38], and are more refractory to the suppressive effect of taxol on MT dynamics *in vitro* [20]. However, in cells these changes have a very limited impact on the sensitivity to taxol, insufficient to establish robust taxol resistance. We also confirmed that other isotypes were not down-regulated as a result of the CRISPRa over-expression, thus, we can conclude that high expression of TUBB3 alone is not sufficient to affect the sensitivity to taxol in RPE cell lines.

Finally, we have examined the functional significance of TUBB3 expression in several breast cancer cell lines that had inherently relatively high expression of this β-tubulin isotype. We find that RNAi-mediated depletion of TUBB3 induced a very minor shift in the sensitivity to taxol in one out of five cell lines analyzed. This finding indicates that TUBB3 expression in breast cancer cell lines is certainly not always linked to taxol resistance. This is exemplified by our observation that TUBB3 levels are dynamically regulated in RPE cells upon short-term exposure of cells to taxol. TUBB3 expression might be rapidly induced in certain cell types upon exposure to conditions of cellular stress. Whether TUBB3 has a functional role in such a stress response remains to be established. All in all, this study, together with previous ones, shows very limited effects of TUBB3 overexpression on the sensitivity to taxol in cultured cell lines.

## MATERIALS AND METHODS

### Cell culture, transfection, and drug treatment

RPE-1, HCT-116, U2OS, CAL51, MDA-MB-231, MCF7, BT20, CAL-120, and SKBR-3 were grown in DMEM (Lonza, Basel, Switzerland), and HCC1937, HCC1187, HCC1806, HCC1395, T47D, BT-549, HCC70, and HS578T were grown in Gibco Advanced RPMI 1640 medium (Fisher Scientific) supplemented with 6% fetal calf serum (Clontech, Mountain View, CA, USA), 50μg/ml penicillin–streptomycin (Invitrogen, Waltham, MA, USA) and 2 mM L-glutamine (Lonza). RPE-11 was obtained from the American Type Culture Collection, and the breast cancer cell lines described in [42, 43]. All cell lines were tested for mycoplasma contamination every three months. siRNA transfections were performed using RNAiMax (Invitrogen) in a reverse transfection protocol following the manufacturer's guidelines. TUBB3 siRNA OTP Human (siTUBB3#8: GCAACUACGUGGGCGACUC, siTUBB3#9: GAAGGAGUGUGAAAACUGC) was purchased from Thermo Scientific and used at a final concentration of 20 nM. Drugs were dissolved in DMSO and prepared at the following concentrations before usage at varying final concentrations as indicated in each figure: Taxol at 100 μM, Vincristine at 1 mM, and Tariquidar at 100 μM.

### Relative survival plots

Cells were plated on 96-well plates (BD Biosciences, Franklin Lakes, NJ, USA) at a starting density of ∼1000 cells per well. Drugs were added the following day. On day 7, plates were fixed for 15 min with 96% methanol at −20°C, stained with 0.1% crystal violet and washed with dH_2_O. Dried plates were scanned and analyzed with ImageJ software (NIH, Bethesda, MD, USA). Cell survival graphs were prepared and IC50 calculations were performed using GraphPad Prism (La Jolla, CA, USA).

### Immunoblot analysis

Cells were lysed in Laemmli buffer. Samples were separated by sodium dodecyl sulfate- polyacrylamide gel electrophoresis and transferred to nitrocellulose membranes, blocked with 4% milk at room temperature, and incubated with primary antibodies at 4°C overnight. The following antibodies were used: anti-TUBB (1:1000; TUB2.1, Sigma-Aldrich), anti-TUBB3 (1:500; TU-20, Millipore), anti-SMC1 (1:1000; A300-055A, Bethyl), anti-HSP90 (1:1000; H114, Santa Cruz), and anti-PGP (1:200; H-241, sc-8313). After incubation with secondary antibody (1:2000, DAKO) at room temperature for 1 hr, the membranes were developed with chemi-luminescence ECL reagent (Amersham, UK) and images were taken with the ChemiDoc XRS+ (Bio-Rad, Hercules, CA, USA). Images were processed and analyzed using ImageJ software (NHI, Bethesda, MD, USA).

### Plasmids

sgRNA sequences for TUBB3 exon3 and ABCB1 were adapted from the genome-wide CRISPRa library [31]. sgRNA sequences for TUBB3 exon1 were selected from a −400 to −50 bp region upstream of the TUBB3 TSS using publically available CRISPR design tool (crispr.mit.edu). sgRNA oligos were cloned into a lentiviral vector (Lentiguide-Puro; Addgene#52963) using the BsmBI restriction site. sgRNA sequences are summarized in Supplementary Table 1.

### Generation of CRISPRa cell lines

RPE cells were co-transduced with viral particles containing SunTag-dCas9-BFP (Addgene# 60910) and scFV-VP69-GFP (Addgene# 60904). After two weeks of culturing, fluorescence activated cell sorting (FACS) was used to select for cells that were both BFP and GFP positive. Monoclonal CRISPRa cell lines were obtained, which were subsequently transduced with viral particles containing pools of sgRNAs targeted at enhancer regions of the ABCB1, TUBB3 exon1 or TUBB3 exon3 loci. Cells were selected for 2 weeks with puromycin to obtain stable polyclonal cell lines for the sgRNA expression.

### RNA isolation and qRT-PCR analysis

Total RNA was extracted from RPE-0, RPE-20 and RPE-CRISPRa TUBB3 (exon3). RNA isolation was performed by using Qiagen RNeasy kit and quantified using NanoDrop (Thermo Fisher Scientific). cDNA was synthesized using SuperScript III reverse transcription, oligo dT (Promega), and 1000 ng of total RNA according to the manufacturer's protocol. Primers were designed with a melting temperature close to 60 degrees to generate 90–120-bp amplicons, mostly spanning introns. cDNA was amplified for 40 cycles on a cycler (model CFX96; Bio-Rad Laboratories) using SYBR Green PCR Master Mix (Applied Biosystems). Target cDNA levels were analyzed by the comparative cycle (Ct) method and values were normalized against actin expression levels. qRT-PCR oligo sequences are summarized in Supplementary Table 2.

### Mass spectrometry

Tubulin bands were excised from the coomassie stained gel, after which proteins were reduced with dithiothreitol and alkylated with iodoacetamide. Proteins were digested with trypsin (mass spec grade, Promega) overnight at 37°C and peptides were extracted with acetonitrile. Digests were dried in a vacuum centrifuge and reconstituted in 10% formic acid for MS analysis. Peptide mixtures (10% of total digest) were loaded directly on the analytical column and analyzed by nanoLC-MS/MS on an Orbitrap Fusion Tribrid mass spectrometer equipped with a Proxeon nLC1000 system (Thermo Scientific) as described previously [39]. Solvent A was 0.1% formic acid/water and solvent B was 0.1% formic acid/80% acetonitrile. Peptides were eluted from the analytical column at a constant flow of 250 nl/min in a 90-min gradient, containing a 74-min linear increase from 5% to 24% solvent B, followed by a 16-min wash at 80% solvent B.

### Mass spectrometry data analysis

Raw data were analyzed by MaxQuant (version 1.5.8.3) [40] using standard settings for label-free quantitation (LFQ). MS/MS data were searched against the human Swissprot database (20,183 entries, release 2017_03) complemented with a list of common contaminants and concatenated with the reversed version of all sequences. Trypsin/P was chosen as cleavage specificity allowing two missed cleavages. Carbamidomethylation (C) was set as a fixed modification, while oxidation (M) was used as variable modification. LFQ intensities were Log2-transformed in Perseus (version 1.5.5.3) [41], after which proteins were filtered for at least three valid values (out of 4 total). Missing values were replaced by imputation based a normal distribution using a width of 0.3 and a downshift of 1.8. Differentially expressed proteins were determined using a *t*-test (threshold: *P* ≤ 0.05) and [x/y] > 1 | [x/y] < −1.

## SUPPLEMENTARY FIGURES AND TABLES


