# Supplementary material for: TUBB3 overexpression has a negligible effect on the sensitivity to taxol in cultured cell lines
Source: Oncotarget. 2017 May 10;8(42):71536–47. doi: 10.18632/oncotarget.17740 (PMC5641069; doi:10.18632/oncotarget.17740)
Supplement: Supplementary file 1 [file oncotarget-08-71536-s001.pdf]

TUBB3 overexpression has a negligible effect on the sensitivity to taxol in cultured cell lines

Supplementary Materials

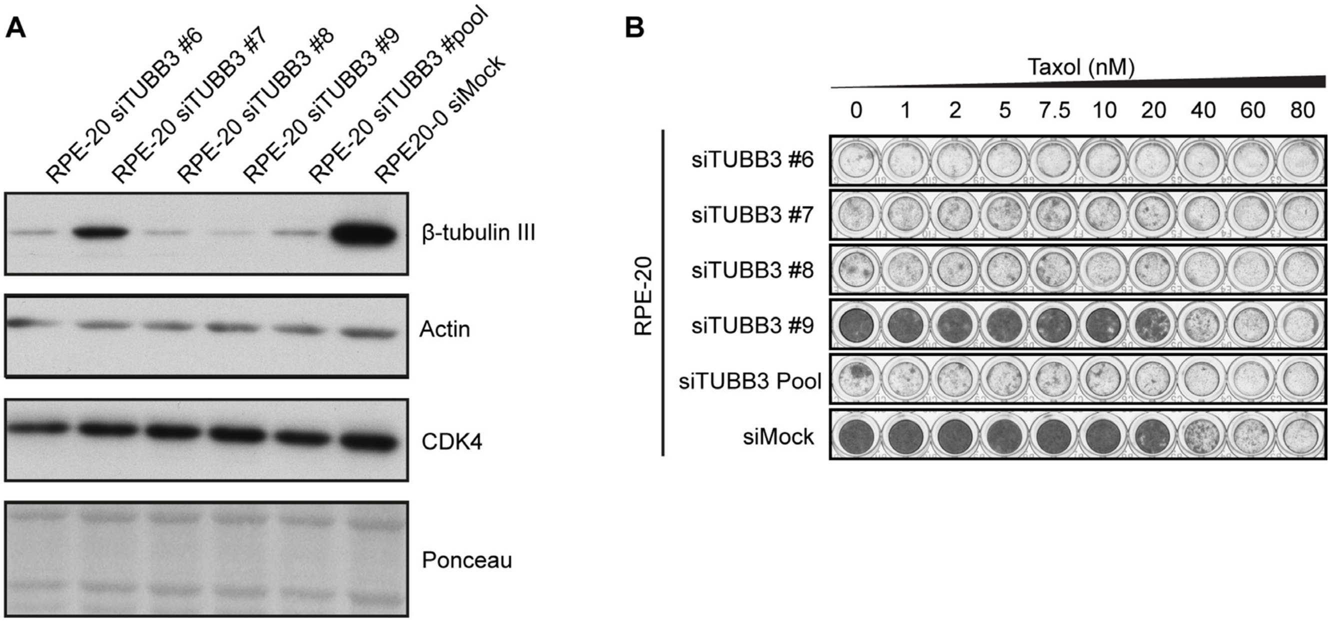

Supplementary Figure 1: βIII-tubulin knockdown does not affect the sensitivity of RPE-20 to taxol. (**A**) Western blot of RPE-20 cells transfected with single siRNAs targeted against TUBB3 for 48 hours. (**B**) Relative survival plots of RPE-20 cells treated with different doses of taxol for 7 days after knockdown of TUBB3 as described in A).

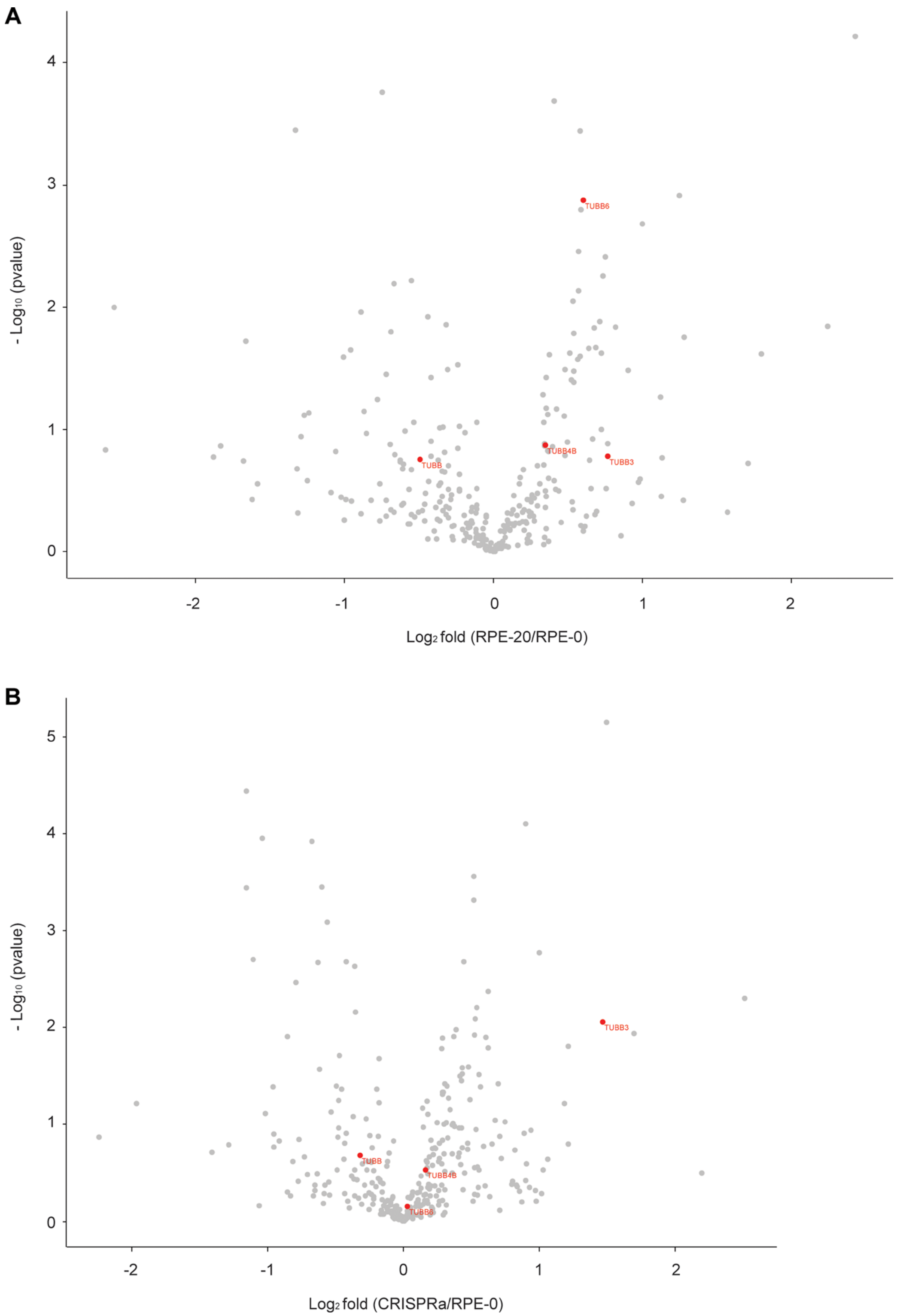

Supplementary Figure 2: Protein quantification of b-tubulin isotypes by mass spectrometry. A volcano plot demonstrating magnitude of the protein comparisons between RPE-0 and RPE-20 in (**A**) and RPE-0 and CRISPRa TUBB3 (exon 3) in (**B**). The vertical axis indicates -log10(*p*-value). The horizontal axis indicates log2 fold change. The b-tubulin isotypes found are labeled in red. Data was obtained from two sample replicates.

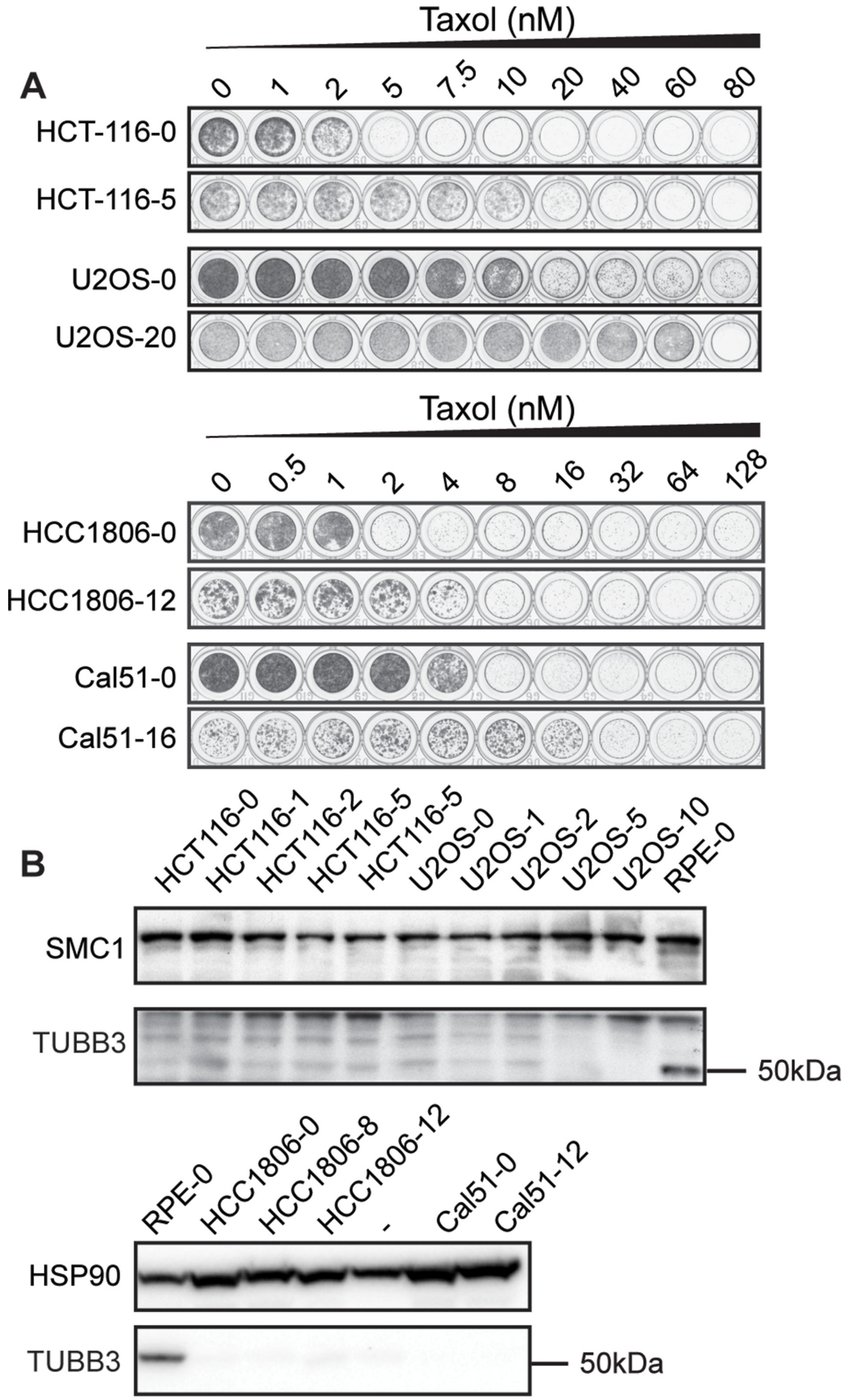

Supplementary Figure 3: Induction of TUBB3 levels is not observed in multiple taxol-resistant cancer cell lines. (**A**) Relative survival plots of taxol-resistant cell lines derived from a colorectal carcinoma (HCT116), an osteosarcoma (U2OS), two triple-negative breast cancer cell lines (HCC1806 and Cal51), and their respective taxol-sensitive parental cell lines. The values added behind the cell lines indicate the concentration of taxol at which the resistant cell lines were grown in (e.g. HCT-116-5 indicates an HCT-116 cell line cultured continuously at 5 nM of taxol). (**B**) Whole cell lysates prepared from the parental and taxol-resistant cancer cell lines showing their TUBB3 levels.

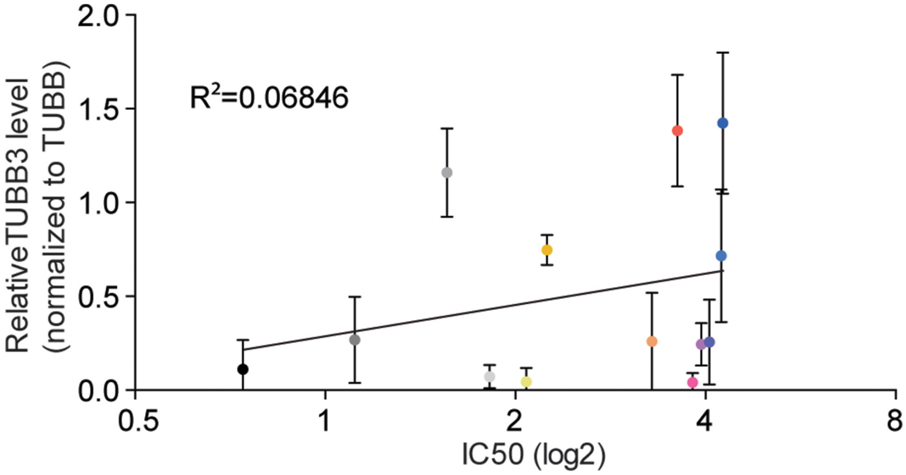

Supplementary Figure 4: Slight correlation between relative TUBB3 levels and IC50. The IC50 values were plotted against the relative TUBB3 levels determined for each breast cancer cell line. TUBB3 levels depicted here are averages from three independent experiments and normalized against whole β-tubulin levels.

Supplementary Table 1: sgRNA target sequences in the putative TSS regions of TUBB3 and ABCB1

TUBB3 exon III	Target sequence	Forward sgRNA oligo	Reverse sgRNA oligo
tubb3-1	ggagctggctgtgggggcgccgtt	caccggagctggctgtgggggcgcc	aaacggcgcccccacagccagctcc
tubb3-2	ggggctgtctcccagaggagcgtt	caccggggctgtctcccagaggagc	aaacgctcctctgggagacagcccc
tubb3-3	gccccgcgcattcgaaagggtt	caccgccccgcgcattcgaaagg	aaaccctttcgaatgcgcggggc
tubb3-5	ggtgcgggttggtctctaaacgtt	caccggtgcgggttggtctctaaac	aaacgtttagagaccaacccgcacc
tubb3-6	gttggtctctaaaccggcgtgtt	caccgttggtctctaaaccggcgt	aaacacgccggtttagagaccaac
tubb3-8	gcggaaccgagagggtagcgtt	caccgcggaaccgagagggtagc	aaacgctaccctctcggttccgc
tubb3-9	ggggatccttggctgcgggaggtt	caccggggatccttggctgcgggag	aaacctcccgcagccaaggatcccc
**TUBB3 exon I**			
tubb3-a	tgcttgaagtttgcgggtggagg	caccgtgcttgaagtttgcgggtggagg	aaaccctccacccgcaaacttcaagcac
tubb3-b	gaaaggagtcccggaaaacgtgg	caccgaaaggagtcccggaaaacgtgg	aaacccacgttttccgggactcctttc
tubb3-d	ggagtgtggaggtcccaaccagg	caccggagtgtggaggtcccaaccagg	aaaccctggttgggacctccacactcc
tubb3-e	agcctagaggaaggacgagcagg	caccgagcctagaggaaggacgagcagg	aaaccctgctcgtccttcctctaggctc
tubb3-f	agctaggacagcgtcaagagtgg	caccgagctaggacagcgtcaagagtgg	aaacccactcttgacgctgtcctagctc
**ABCB1**			
abcb1-1	gtagctcctcctctggtactgtt	caccgtagctcctcctctggtact	aaacagtaccagaggaggagctac
abcb1-2	gtgtttatcccagtaccagagggtt	caccgtgtttatcccagtaccagagg	aaaccctctggtactgggataaacac
abcb1-3	gaggagctacatgaactaaggcgtt	caccgaggagctacatgaactaaggc	aaacgccttagttcatgtagctcctc
abcb1-4	gctacatgaactaaggcaggcgtt	caccgctacatgaactaaggcaggc	aaacgcctgccttagttcatgtagc
abcb1-5	gcactaatcagtgaaaacccagtt	caccgcactaatcagtgaaaaccca	aaactgggttttcactgattagtgc
abcb1-6	gaaaacccaaggataagtttgtt	caccgaaaacccaaggataagttt	aaacaaacttatccttgggttttc
abcb1-7	gataagtttgggtggaggaaggggtt	caccgataagtttgggtggaggaaggg	aaacccccttcctccacccaaacttatc
abcb1-8	gtgatctttttgctaaggtgtgtt	caccgtgatctttttgctaaggtgt	aaacacaccttagcaaaaagatcac
abcb1-9	gagttacatggcttagggatgtt	caccgagttacatggcttagggat	aaacatccctaagccatgtaactc
abcb1-10	gttgagaagtttagccagaatgtt	caccgttgagaagtttagccagaat	aaacattctggctaaacttctcaac

Supplementary Table 2: β-tubulin isotypes and ABCB1 RT-qPCR oligos

	Forward oligo	Reverse oligo
**TUBB/tubulin beta class I**	GCCTTCCTCCACTGGTACAC	GCCTCCTCACCGAAATCCTC
**TUBB1/ tubulin beta 1 class VI**	GCTTCCAGATCGTCCACTCC	GTCCGACACCTTGGGAGAAG
**TUBB2A/tubulin beta 2A class Iia**	CTGAAGATGTCGGCCACCTT	CGGTGAACTCCATCTCGTCC
**TUBB2B/tubulin beta 2B class Iib**	CTGAAGATGTCGGCCACCTT	CGGTGAACTCCATCTCGTCC
**TUBB3/tubulin beta 3 class III**	GCCTCAAGATGTCCTCCACC	CGGTGAACTCCATCTCGTCC
**TUBB4A/tubulin beta 4A class Iva**	GACTCTGGAAACCGCACCTT	TCAGAGGGAGGGGTCAAACA
**TUBB6/tubulin beta 6 class V**	TCCACCTTCATCGGCAACAG	TTTCATCCATGCCCTCACCC
**TUBB8/tubulin beta 8 class VIII**	GCCTTCCTCCACTGGTACAC	CTCCTCCTCGGCATACTCCT
**ABCB1**	CAGTTGAGTGGTGGGCAGAA	GCCTTATCCAGAGCCACCTG
